# A method for rapid quantitative assessment of biofilms with biomolecular staining and image analysis

**DOI:** 10.1007/s00216-015-9195-z

**Published:** 2015-12-07

**Authors:** Curtis Larimer, Eric Winder, Robert Jeters, Matthew Prowant, Ian Nettleship, Raymond Shane Addleman, George T. Bonheyo

**Affiliations:** Pacific Northwest National Laboratory, Battelle for the USDOE, PO Box 999, MSIN P7-50, Richland, WA 99352 USA; Marine Sciences Laboratory, Pacific Northwest National Laboratory, 1529 W. Sequim Bay Road, Sequim, WA 98382 USA; Swanson School of Engineering, University of Pittsburgh, Benedum Hall, 3700 O’Hara Street, Pittsburgh, PA 15261 USA

**Keywords:** Biofilm, Biofouling, Image analysis, Biomolecular stain, Biofilm growth intensity

## Abstract

The accumulation of bacteria in surface-attached biofilms can be detrimental to human health, dental hygiene, and many industrial processes. Natural biofilms are soft and often transparent, and they have heterogeneous biological composition and structure over micro- and macroscales. As a result, it is challenging to quantify the spatial distribution and overall intensity of biofilms. In this work, a new method was developed to enhance the visibility and quantification of bacterial biofilms. First, broad-spectrum biomolecular staining was used to enhance the visibility of the cells, nucleic acids, and proteins that make up biofilms. Then, an image analysis algorithm was developed to objectively and quantitatively measure biofilm accumulation from digital photographs and results were compared to independent measurements of cell density. This new method was used to quantify the growth intensity of *Pseudomonas putida* biofilms as they grew over time. This method is simple and fast, and can quantify biofilm growth over a large area with approximately the same precision as the more laborious cell counting method. Stained and processed images facilitate assessment of spatial heterogeneity of a biofilm across a surface. This new approach to biofilm analysis could be applied in studies of natural, industrial, and environmental biofilms.

**Electronic supplementary material:**

The online version of this article (doi:10.1007/s00216-015-9195-z) contains supplementary material, which is available to authorized users.

**Graphical abstract:**
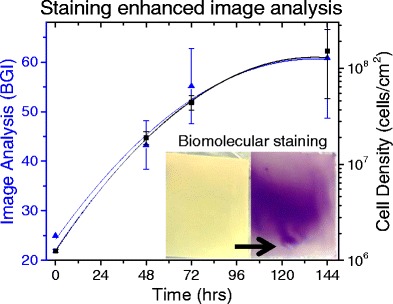
A novel photographic method was developed to quantify bacterial biofilms. Broad spectrum biomolecular staining enhanced the visibility of the biofilms. Image analysis objectively and quantitatively measured biofilm accumulation from digital photographs. When compared to independent measurements of cell density the new method accurately quantified growth of *Pseudomonas putida* biofilms as they grew over time. The graph shows a comparison of biofilm quantification from cell density and image analysis. Error bars show standard deviation from three independent samples. Inset photographs show effect of staining

## Introduction

In natural aqueous environments, man-made interfaces are subjected to accumulation of unwanted biological material in the form of stable bacterial biofilms [1]. It is difficult to accurately quantify early-stage development of biofilms, particularly in situ [2], because they are typically composed of diverse groups of microscopic organisms and other organic material that form heterogeneous, soft, and often transparent structures [3]. In this work, a versatile photographic method was developed to make the heterogeneous structure of biofilms more visible. A broad-spectrum mixture of biomolecular stains was used to highlight primary and secondary metabolite components that make up the biofilm, and image analysis was used to quantify the overall amount of biofilm growth visible in an image. This new analytical approach will be discussed in the context of existing methods and in relation to potential applications in biomedical, industrial, and marine settings.

Biofilms form when microbes settle on a surface and discharge a sticky matrix of polymeric substances that protect them and eventually attract or trap more or larger fouling organisms [4, 5]. Biofilms and biofouling are harmful even in the earliest stages: thin layers of biofilm on medical implants routinely lead to full-fledged infections [6], and an increase in roughness of a ship’s hull by as little as 10 μm can increase drag and affect fuel efficiency [7]. Fouling inhibits flow through industrial filters [8], exacerbates corrosion [9], reduces heat transfer efficiency [10], persists in water distribution networks [11], and otherwise permeates the built environment with deleterious effects. Often, fouling occurs in places that are not suited to traditional sanitary laboratory testing so quantifying biofilm growth in the environment is a challenge.

There are several American Society for Testing and Materials (ASTM) standards for the assessment of biofouling on marine antifouling coatings [12–15]. However, these standards have limited applicability, require long-term data collection (up to 2 years), and are only semi-quantitative because they rely on subjective estimates of areal coverage based on visual inspection and on counting organisms of various fouling species (e.g., barnacles, oysters/mussels, tubeworms, algae, etc.). Since the methods are based on visual inspection, it is not possible to quantitatively evaluate the development of early-stage fouling. Subtle differences in this soft, transparent or semi-transparent, heterogeneous film of microorganisms cannot be distinguished with the naked eye, yet may serve as an important predictor of the development of fouling in the long term [16]. Moreover, the conditioning film, biofilm, or slime layer often covers the full area of a sample surface but it does not do so evenly, so areal coverage can be a misleading measurement that does not accurately represent the progress of fouling development.

By contrast, there is a strong collection of ASTM standards for the evaluation of biofilm formation [17–21]. These test methods serve as a model for improved quantification of biofouling; however, they are exclusively for laboratory evaluation and require specialized bioreactors. These methods are not suited to field evaluation and can be time and labor intensive. Additionally, the reactors have a limited range of flow rates (from static to ∼7 mL min^−1^), hold a limited number of samples, and cannot easily be adapted for use with multispecies biofilm communities.

Optical biosensors have been employed to analyze biofouling and biofilm formation including bright-field light microscopy, confocal microscopy, fluorescent microscopy, or bulk optical techniques like fluorescence, reflectance, and absorbance [22]. These techniques offer a high level of detail such that individual bacterial cells can be seen at high magnifications; however, the utility of optical images is often limited by a narrow field of view that cannot capture the heterogeneous and topologically diverse nature of bacterial biofouling (see Electronic Supplementary Material (ESM) Table S1 for a table comparing biofilm imaging methods). For example, confocal laser scanning microscopy can reconstruct biomass distribution in 3-D [23] but only for biofilms of a limited thickness and opacity. Measuring large areas (>1 mm^2^) of biofilms is time consuming and in some cases impossible. Because the field of view for microscopy methods is typically limited, complex random sampling techniques and statistical analysis must be used to avoid biased results [24, 25]. Quantitative information can be obtained from microscope images via automated image analysis [26, 27]. COMSTAT is a standard image analysis program for biofilm quantification that uses binary thresholding to separate biomass from interstitial space [28]. The program analyzes image stacks recorded by confocal microscopy. Binary thresholding has been used to evaluate biomass distribution in bacterial biofilms [29], but there are many limitations when the images do not have adequate separation of grayscale intensities between the foreground and background [30].

Of note is the frequent use of staining techniques to enhance contrast in light microscopy [31]. Biomolecular stains selectively adhere to specific cellular or biological components (e.g., proteins, nucleic acids) and can be used to enhance fluorescence [32], assist in the identification of microorganisms or tissues [31], and identify live or dead cells [33]. Combinations of dyes can be used to differentiate and enhance features within images of biological samples [34]. A common stain that has been used to quantitatively assess biofilms is crystal violet, which was noted for its low cost and high reproducibility in a comparison of standard methods [35]. Overall, use of dyes and staining can enable or enhance quantitative aspects of analytical biochemistry.

In this work, a simple way to quantify the early stages of biofouling and biofilm formation is presented. This method enables measurement of fouling over the entire area of a surface of interest. A select combination of dyes was used to stain major components of biofilm growing on sample surfaces. The staining process was designed to enhance biofilm contrast in digital photographs. Image analysis was performed on biofilm photographs using a new multilevel thresholding algorithm, and this algorithm was compared to existing image analysis methods. This novel approach to biofilm quantification offers significant advantages over existing standard methods that are complex, inaccurate, or lab confined. This method of analysis will be useful for rapid analysis of biofilm formation and biofouling in biomedical, industrial, and marine settings.

## Methods and materials

### Biofilm culture

Sample coupons were exposed to a liquid culture of *Pseudomonas putida* (ATCC 39169) bacteria, then stained, photographed, and analyzed using an image analysis algorithm program written for the purpose. In parallel, identical coupons were analyzed by a standard method: cell density of the biofilm was measured after removing the cells by sonication and dispersing them in solution. Square 2.5- by 2.5-cm coupons of FR4 fiberglass (McMaster-Carr, Los Angeles, CA) were placed in petri dishes with 25 mL tryptic soy broth (BD Biosciences, San Jose, CA). Each dish had two coupons, one for staining and image analysis and one for cell density measurements. The dishes were inoculated with 100 μL *P. putida* and left covered in static and ambient conditions (∼20 °C) for up to 6 days (144 h). Additional tryptic soy broth (TSB) was added after 2 days to prevent dehydration of the remaining samples. At four time points (0, 48, 72, and 144 h), triplicate samples were removed and analyzed.

### Biomolecular staining

One coupon from each dish was gently removed and fully submerged for 1–2 s in a bath of phosphate-buffered saline (PBS) to remove unbound or unattached fouling material. Staining mixture (100 μL) was applied to the samples with a pipette over the full area of the sample. The stain contained 100 mL of 0.1× concentration phosphate-buffered saline (PBS) at pH 7.4 with 0.1 g of erythrosine B (Thermo Fisher Scientific, Waltham, MA), 0.2 g KeyAcid Rhodamine (Keystone Analine Corporation, Chicago, IL), and 0.3 g Coomassie Brilliant Blue G-250 (Thermo Fisher Scientific) mixed in. PBS 1× concentration is comprised of NaCl (8 g L^−1^), KCL (0.2 g L^−1^), Na_2_HPO_4_ (1.44 g L^−1^), and KH_2_PO_4_ (0.24 g L^−1^) with each component purchased from Thermo Fisher Scientific. The composition of this stain mixture will be discussed in more detail below. After the stain was applied, the sample was immediately submerged gently in PBS again for 1–2 s to remove excess stain. Control coupons were pre-rinsed, “stained” with 100 μL of PBS, and dip rinsed in PBS in the same manner as other coupons. See below for further processing of control samples to quantify cell density.

At the conclusion of the staining procedure, each stained sample was placed in a clean dish below a digital camera (Panasonic DMC-LX3) positioned with a stand. Lighting for the photographs was controlled using the overhead lights in a biosafety cabinet in order to be consistent for each set of samples. Bright diffused lighting was preferred to avoid shadows, glare, and reflections on the samples. In later experiments done outside the biosafety cabinet (not shown here), LED lighting panels (LimoStudio model AGG1089) were used to provide bright diffused lighting. The camera was operated in manual mode with f/2, 1/60 s exposure, ISO 400, 5 mm focal length and no flash. The photos were recorded in RAW format for later processing.

### Quantifying biofilm growth—control

After undergoing the staining procedure (with PBS in place of stain), triplicate samples for each time point were placed in conical 50-mL centrifuge tubes. Exactly 15 mL of PBS with 0.05 % Tween 20 (Sigma-Aldrich) was added to each tube—enough to fully cover the sample. The sample tubes were placed in a sonicating bath filled with water for 30 min to remove biofilm from the surface and disperse it into the buffered saline. After sonication, the biofilm was further dispersed with a vortex mixer prior to analysis. Three 1-mL samples were pipetted from each test tube into disposable spectroscopy cuvettes. The optical density at 600 nm (OD_600_) was measured for each sample using a UV-visible spectroscopy system (Agilent 8453). Optical density was chosen over viable cell count as it provides a more accurate measure of the total number of cells (live, dead, and non-culturable) and also takes into account cell size (i.e., that large cells have a greater contribution to optical density and to biomass on the surface of a coupon). Optical density was measured in absorbance units (AU) and compared to a reference sample with PBS and Tween 20 but no bacteria. In the range 0–1 AU, optical density is roughly proportional to cell density with 3.9 × 10^8^ cells mL^−1^ per unit AU [36]. This relation was used to calculate the number of cells dispersed in the solution (cells mL^−1^) and that was converted to areal density (cells cm^−2^) of biofilm on the surface of the coupon (counted as both sides of the 2.5- by 2.5-cm coupon, excluding edges). Areal cell density was used as a control against which the image analysis algorithm presented in this work was compared.

### Quantifying biofilm growth—image analysis

Coupons stained by the procedure described above appear darkest wherever the biofilm is thickest and most developed. Therefore, color intensity can serve as the basis for quantitative image analysis. A standard image analysis technique is to establish a threshold to delineate foreground (biofilm) from background (clean). The threshold value can be chosen manually or algorithmically, and this technique works well when an image contains two distinct regions. Otsu’s method of thresholding is one of the most referenced algorithmic techniques [37, 38]. Otsu’s method reduces a grayscale image to a binary image by assuming pixel values form a bimodal histogram and can therefore be separated into two classes by setting a threshold that minimizes intra-class variance. However, this method gives best results when there are approximately the same number of pixels in each class and the color or gray levels of the foreground and background are significantly different and non-overlapping. Though simple binary thresholding is commonly used in biological microscopy image analysis [39–43], these techniques are inadequate for accurate macroscale quantification of biofilms because the early stages of fouling result in subtle, indistinct changes in the appearance of the surface.

**Fig. 1:**
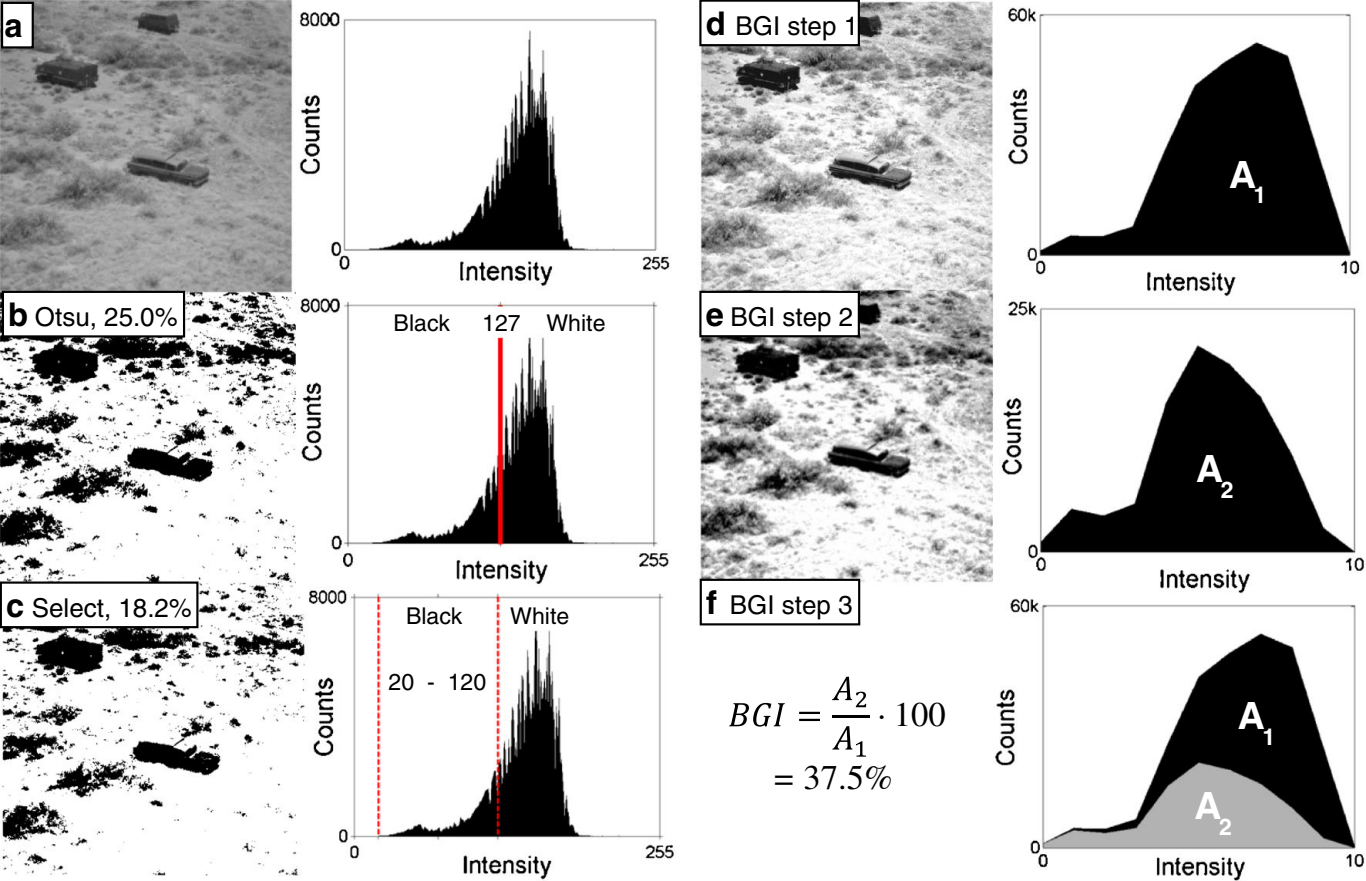
Images and corresponding intensity histograms subject to quantification by image analysis. The goal in processing this image is to separate high-contrast signal from background. The original standard image (**a**) is converted into binary images by two standard methods—Otsu’s thresholding (**b**) and manually selected thresholding (**c**). The signal is quantified by calculating the percentage of the area covered by *black pixels*. Both methods can easily separate the dark objects from the surrounding landscape, but both lose valuable information about the intensity of the background and underestimate the true signal. **d**–**f** illustrate the steps for the analysis algorithm used in this work—BGI. The first step (**d**) applies a multilevel threshold to the image (*N* = 10), which also stretches the histogram to enhance contrast. In the second step (**e**), a ramp filter is applied to the histogram. To calculate BGI (**f**), the area under the filtered histogram (*A*_*2*_) is divided by the area under the original histogram (*A*_*1*_). This algorithm can distinguish high-contrast objects and medium-contrast objects from the background. The relative importance of high-contrast objects (which in this example could represent macrofouling) is reflected in the calculation of the final BGI value. Low-contrast features (i.e., representing microfouling) are weighted less

Therefore, a new method of image analysis using multilevel thresholding was developed to rapidly measure biofilms in digital photographs. The analysis evaluates biofilm growth intensity (BGI) on each coupon and assigns a value between 0 and 100 that represents an absolute score for biofilm intensity over the entire coupon. Measuring BGI allows direct and quantifiable comparison of biofilm growth on different coupons. BGI is not just a measure of areal coverage of the biofilm: it also accounts for density and thickness of surface growth. BGI was compared to two existing binary thresholding techniques—Otsu’s method and a manually selected threshold. These image analysis methods are illustrated in Fig. 1 with a standard image that was selected from a database of digitized images maintained by the Signal and Image Processing Institute at the University of Southern California (http://sipi.usc.edu/database/). Though this image is not a photograph of a biofilm, it serves as a representative example because it has elements that are easy to distinguish with traditional binary threshold analysis (separation of the dark cars from the ground) and elements that are difficult to distinguish such as the difference between the ground and plants.

Figure 1a shows a standard grayscale image and a histogram of the frequencies of each grayscale intensity level. The histogram has a strongly uneven bimodal distribution. Otsu’s method (Fig. 1b) assigns all pixels with intensity below the threshold to one class (black) and all pixels above the threshold to the other (white). The resulting binary image has separated the darkest elements of the original photo from the background. The signal constitutes 25.0 % of the area of the image. Figure 1c illustrates a similar binary threshold technique, though in this case the threshold was manually selected. As expected, this method attributes more of the image to background, probably because of selection bias, and the areal coverage was just 18.2 %.

Figure 1d–f shows three of the steps involved in the BGI algorithm. First, a multilevel threshold is applied to the image (Fig. 1d). The purpose of the multilevel threshold is to enhance visibility of the full spectrum of biofilm growth. This threshold is similar to Otsu’s method in that it minimizes intra-class variance between groups set by the threshold. Instead of just one threshold (and two resulting groups), ten thresholds divide the image into 11 groups. The threshold levels can be determined in multiple ways—typically through recursive optimization [44–47]. The multilevel threshold also has the effect of stretching the image’s histograms to enhance contrast. The second step is to apply a simple linear ramp function to the histogram(s) of the thresholded image. The ramp filter enhances the relative importance of high-intensity values in each data set (Fig. 1e). Pixels in the first group were multiplied by 0, the second group by 1/11, the third group by 2/11, and so on, with the last group multiplied by 1. Ramp filters are often used for medical images such as PET and CT scans [48]. In this case, the filter is linear and therefore would not artificially introduce a non-linear trend. See ESM Fig. S1 for examples of the effects of the ramp filter.

The final image created by the BGI algorithm retains greater fidelity to the original image. The histograms shown in Fig. 1f are then used to calculate a single number that is representative of biofilm in the image. The filtered histograms of samples with thick biofilms are not significantly changed by the ramp filter; on the other hand, the histograms from relatively clean samples will be heavily filtered. Comparing the area under the filtered histogram to the area of the thresholded histogram is a simple way to measure how dark or light an image is overall and, thus, how much biofilm it has. This was done by dividing the area of the filtered histogram (A_2_) by the area of the original histogram (A_1_, which is the total number of pixels in the image) to give a single number that would be representative of total biomass. This value was named the biofilm growth intensity (BGI) and is written as a percentage.

However, note that BGI does not measure the percent of area covered by biofilm. A BGI value of 100 represents an image with 100 % of pixel intensity values in the darkest threshold group. Likewise, BGI of 0 would be made up of pixels only in the brightest group. ESM Fig. S1 shows additional examples of BGI calculation over a range of values. In summary, the BGI algorithm consists of applying a multilevel threshold to create a simplified and stretched histogram, applying a ramp filter to the resulting histogram, and calculating BGI from the ratio of the areas of these two histograms.

A Matlab software package was written to compile the multistep BGI analysis process (see ESM Fig. S2 for a flow chart of this process). Briefly, this software package prompts the user to select a sample image to import. The user selects an area of each sample to be analyzed that does not include markings, any holes used to secure samples, or sample edges. Since the image was captured with a CCD sensor with red, green, and blue pixels, color information can be extracted from the images by separating the color channels of the 32-bit full-color image. The BGI algorithm is applied to histograms of each color. Converting the color image to grayscale can also serve as an overall average. It was hypothesized that the colors could differentiate features within the biofilm while the grayscale image serves as an overall average. Analysis of the color channels was used to accurately identify biomass as opposed to shadowing due to the texture of the coupon or the biofilm. The Matlab script and several example images can be found at https://github.com/curtislarimer/Biofilm-Growth-Intensity.

Staining prior to image analysis was essential to accurately measure biofilm growth using the BGI algorithm. Without staining, changes in the amount of biofilm on the surface would not result in an appreciable difference in color or grayscale intensity. The stains were selected to target and attach to living organisms and other organic matter that is present in biofilms. The staining process resulted in digital images with color intensity representative of the amount of biofilm present.

### Statistical comparison of biofilm quantification methods

The extent of biofilm growth seen in digital images was quantified using Otsu’s thresholding, manually selected thresholding, and the BGI algorithm presented in this work. All three methods of image analysis were compared to the areal cell density. Comparisons were made using three pairs of identical coupons at each of four exposure time points. The Pearson correlation coefficient (*r*) was determined for each comparative pair. Also, the slope of a simple linear regression (*m*) was calculated between data pairs. Best fit between two sets of data occurs when *r* and *m* are both nearest to 1. The coefficient and slope were multiplied together to give a single measure of best fit (0 to 1). All image analysis methods were ranked to determine which best fit the trend observed in surface cell density.

## Results

An analytical method was developed to quantitatively determine biofilm growth on sample coupons. The method consisted of three simple steps: biomolecular stain was applied to the sample, the sample was photographed, and the digital photograph was analyzed digitally. The entire process can be completed in minutes, though it was typical to stain and photograph multiple samples and perform image analysis subsequently. Staining the samples was essential for successful subsequent image analysis. Biomolecular dyes were selected to highlight several components of surface-attached biomass. As noted above, the stain mixture contained erythrosine B, Rhodamine, and Coomassie Brilliant Blue (see ESM Fig. S3). Erythrosine B is a cherry pink organoiodine compound that adheres to phosphoproteins and is used to identify microorganisms, rhodamine is a xanthene dye used to detect nucleic acids, and Coomassie Brilliant Blue G-250 is a triphenylmethane dye used to stain proteins [49]. It was found in preliminary testing that this group of stains broadly attached to biomass without staining the underlying substrate (a problem observed with crystal violet). Individual stains did not provide sufficient enhancement of contrast and did not uniformly attach to the diverse components that make up bacterial biofilms.

**Fig. 2:**
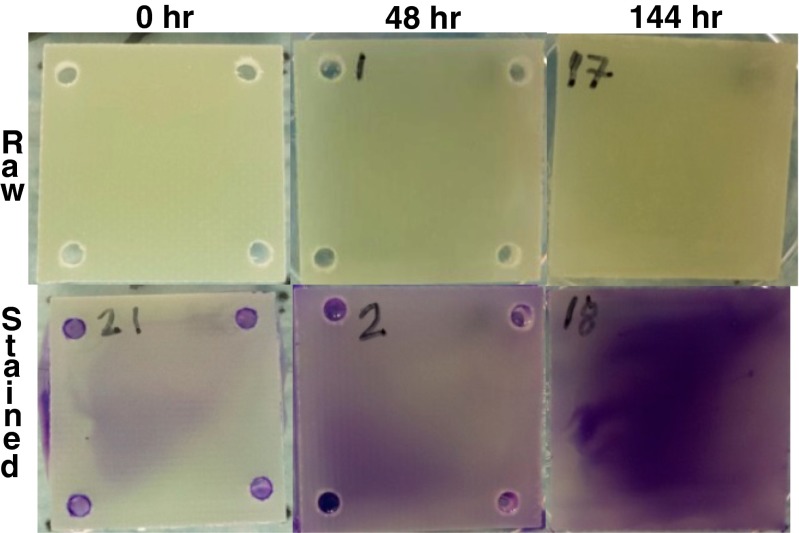
Raw and stained images of fouled coupons at 0, 48, and 144 h. Differences in fouling on each coupon are subtle in the raw images. Coupons that were stained show an obvious increase in fouling over time. The size of each coupon is 2.5 by 2.5 cm

The first row of Fig. 2 shows representative photographs of biofilm samples from select time points. Although biofilm growth was expected to increase with time, differences in the raw photographs of unstained coupons are very subtle, and samples exposed for 48 and 144 h are only slightly darker than the original fiberglass coupon. The slight change in the images over time appears uniformly distributed over the area of the coupon. As seen in the second row of Fig. 2, staining has enhanced visibility and contrast of biofilm growth across the area of each coupon and between the different time points. The biofilms are clearly non-uniform and the increase in biofilm growth from 0 to 144 h is obvious.

**Fig. 3:**
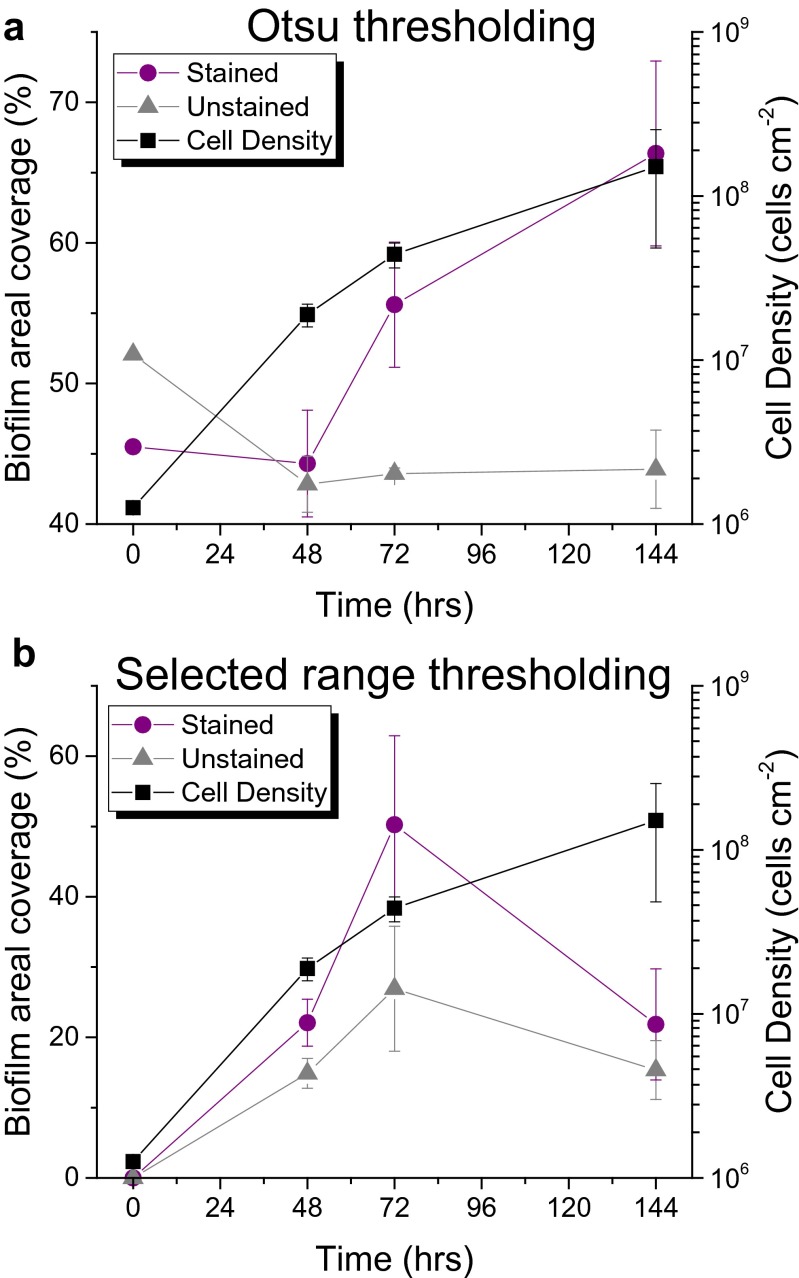
Comparison of cell density to areal coverage of biofilm by **a** Otsu’s thresholding method and **b** a manually selected threshold range. Data is shown for stained (*purple*) and unstained (*gray*) coupons. Cell density rises exponentially, as expected. Neither binary thresholding technique accurately matches cell density data. *Error bars* show standard error from triplicate measurements

Images of stained and unstained coupons like those seen in Fig. 2 were analyzed using two simple bimodal thresholding techniques. These image analysis algorithms divided each image into two classes according to a threshold that was set manually or determined by Otsu’s method (see example images in ESM Fig. S4). The area covered by biofilms was calculated from the resulting binary images and was compared to cell density on each surface as measured by the optical density of a cell suspension created by removing the surface-attached bacteria. As seen in Fig. 3, cell density increases exponentially with time as is expected for bacterial growth. Otsu’s method (Fig. 3a) could not successfully measure areal coverage on unstained coupons because the nearly uniform images do not have distinct bimodal distributions of pixel intensity. In each case, Otsu’s method merely resulted in biofilm areal coverage near 50 %. Otsu’s method fared better with stained images. A clearly increasing trend is seen with time. However, even at the beginning of the experiment, the areal coverage was greater than 40 %, there was no increase between 0 and 48 h, and variation among the triplicate samples was high.

In Fig. 3b, it can be seen that selected range thresholding did not match cell density over time. This method accurately resulted in an areal coverage near 0 at the beginning of the experiment and an increasing trend for both stained and unstained images through 72 h. However, at 144 h, this thresholding technique resulted in a sharp decline and drastically underestimated biofilm growth. As a result, neither binary thresholding technique resulted in a satisfactory trend in areal coverage that matched the increase in cell density. Statistical analysis presented below confirms this conclusion.

**Fig. 4:**
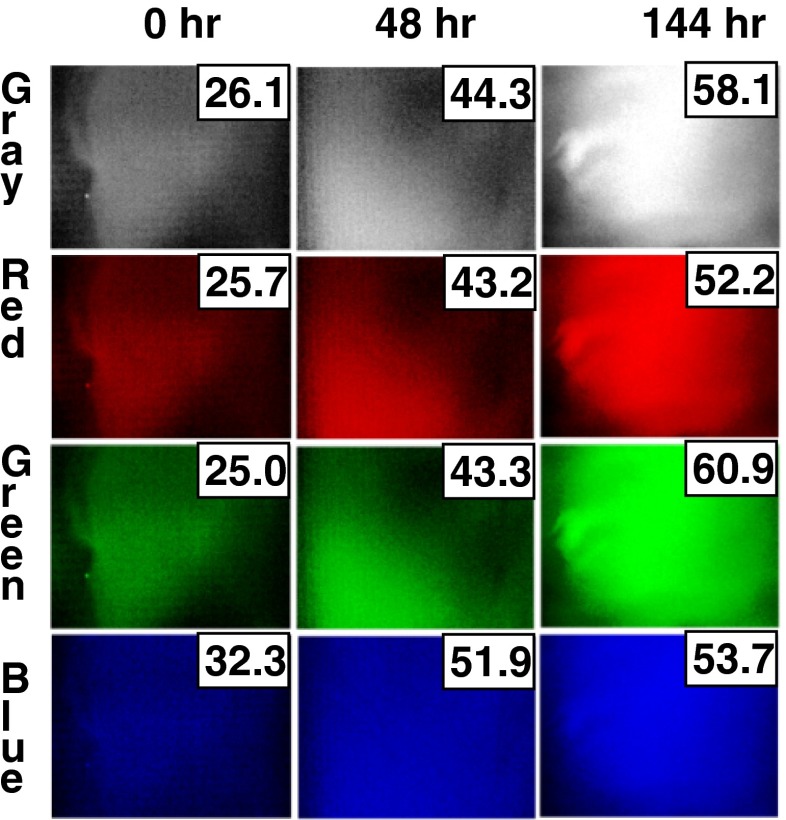
BGI image analysis of stained coupons seen in the second row of Fig. 2 produces four images for *gray*, *red*, *green*, and *blue. BGI values* for each image are overlaid. Each *color* shows an increase in BGI from 0 to 144 h. *Gray* and *green images* appear to retain finer detail in the image and have distinct BGI values

Because of the lack of an adequate image analysis method to quantify biofilm growth, a new algorithm was developed to measure biofilm growth intensity (BGI). Figure 4 shows example images created during BGI analysis of the stained coupons seen in Fig. 2. The BGI algorithm was used to analyze the grayscale image as well as each of the three color channels of an image. Clear changes in the color intensity can be seen as biofilm accumulates and grows over time. It was expected that analysis of separate color channels could distinguish distinct aspects of the biofilm (e.g., microorganisms, extracellular matrix, etc.). However, no major differences were observed, perhaps because at this early stage of biofilm development the different phases are well intermixed at the scale observed. Interestingly, blue and red images appear saturated at 144 h while gray and green images more clearly show the distinct macroscopic morphology of the biofilms. The BGI algorithm extracted a quantified value of biofilm growth from visually observable trends seen in Fig. 4. These images are easy to visually inspect and provide a platform for further unbiased quantification.

**Fig. 5:**
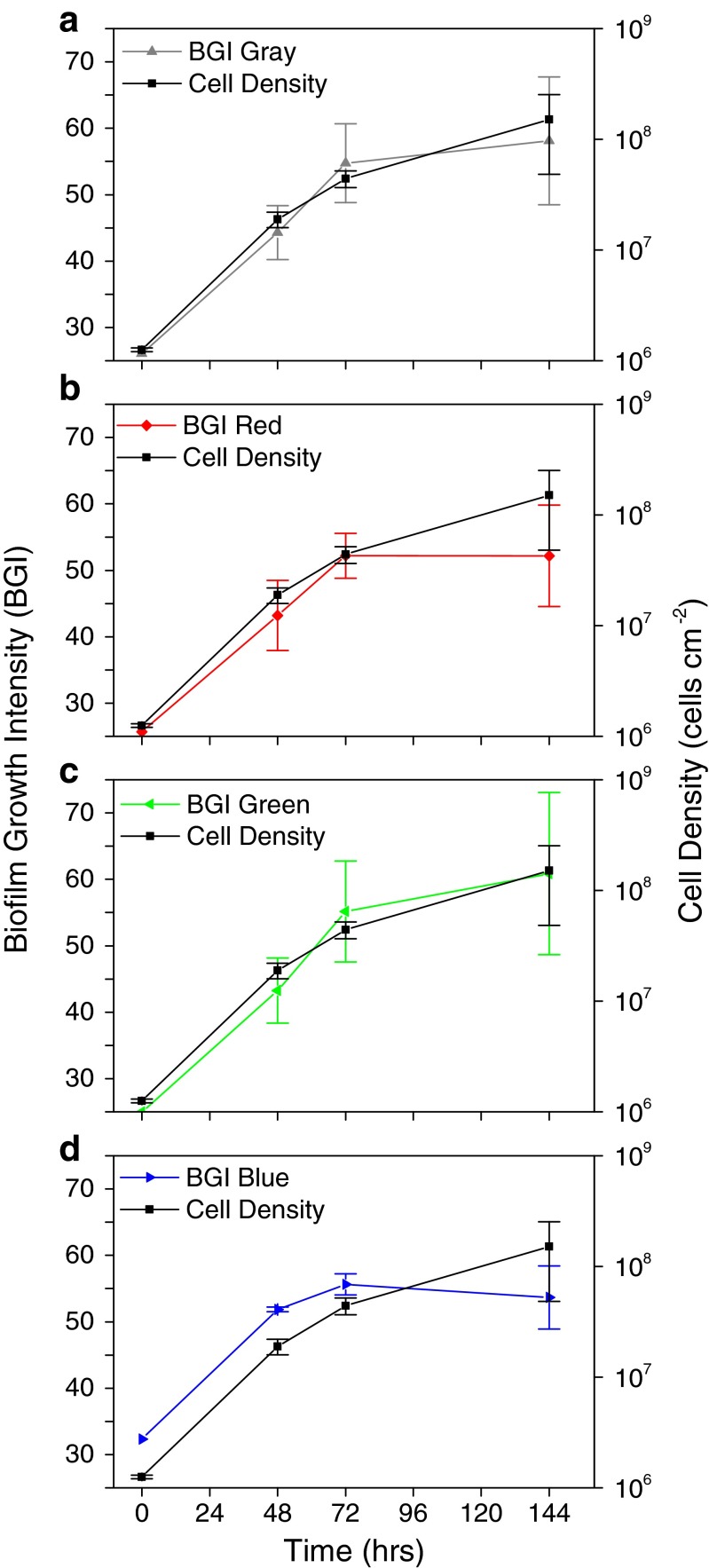
Comparison of BGI measure of biofilm growth to cell density measured by optical density. BGI is shown on a linear scale (*left*) and cell density is shown on a log scale (*right*). Plots show well-matched data from **a** grayscale, **b** red, **c** green, and **d** blue channels of full-color digital photos. *Error bars* show standard deviation from three independent samples in all cases

Figure 5 compares the biofilm growth intensity to cell density measured over time for each of the color channels of digital photographs. BGI data matches the trend observed for cell density. BGI measured from a grayscale image and from the green channel (Fig. 5a, c) appear to fit better than data from the red and blue channels (Fig. 5b, d). The rise in BGI leveled off in the red and blue channels after 72 h likely because those color channels were saturated more quickly by the purple-hued stain. BGI data appears to be well correlated with cell density on the surface. An increase in BGI by approximately 15 corresponds to a 1 log increase in cell density on the surface. The standard deviation of BGI measurements from triplicate samples was approximately 5 (though this increases with the magnitude of the measurement), so BGI can accurately predict cell density to within ∼1/3 of a logarithmic unit change in cell density, which is approximately the same precision as the optical density method of measuring biofilm growth.

**Fig. 6:**
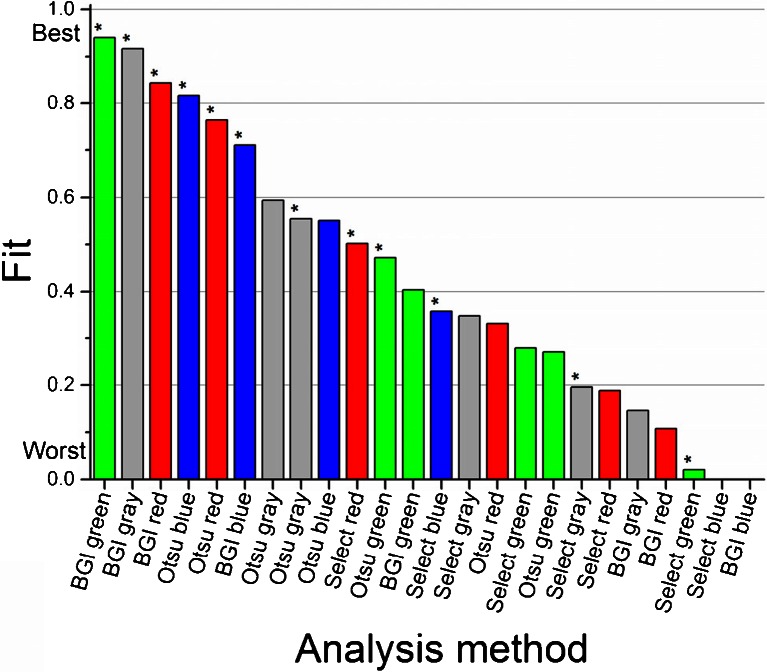
Ranking of image analysis methods from best to worst fit with independently measured cell density. Image analysis algorithms applied to coupons that were stained are indicated by a *star* (*). The *color* of each *bar* corresponds to the color channel analyzed (*red*, *green*, and *blue*) with *gray* indicating a grayscale version of the full-color image. The BGI algorithm ranked better than Otsu’s method or manually selected thresholding. Staining uniformly improved fit of image analysis data and was essential for the BGI algorithm

Each of the algorithms was tested on each of the color channels of stained and unstained coupons and compared to cell density data to determine if experimental models fit well. In Fig. 6, all the methods of analysis are ranked from best to worst fit. A table of statistical values is available in ESM Table S2. Data collected from stained coupons was almost uniformly ranked better than data from unstained coupons: nine of the top 12 analyses benefitted from enhanced contrast in images with staining. Correlation between image analysis and cell density measures of biofilm growth was strongest when the BGI algorithm was applied to stained images. BGI analyses of green, gray, and red data were the three highest ranked methods of analysis. Neither Otsu’s method nor the manually selected threshold fit well with the independent measurement of cell density. Interestingly, the BGI algorithm alone ranked poorly; however, the combination of the staining procedure with the novel BGI image analysis resulted in robust quantification. Examples of manipulated histograms can be seen in ESM Fig. S5. The BGI algorithm correlated well primarily because the algorithm accurately quantified a broader range of biofilm growth from initiation (when no bacteria was present) to maturity (when the stained biofilm was dark and widespread).

## Discussion

A simple, rapid assay was developed to quantify early stages of biofilm growth. The method requires only a photograph of a stained surface that is then subject to digital image analysis using a new image analysis algorithm for quantification. The entire process can be completed in minutes. Visibility of the biofilm was enhanced with the use of a mixture of biomolecular stains selected to provide broad-spectrum indication of biomass. Traditional image analysis algorithms using binary thresholding did not result in data that matched the increasing trend in cell density on the surface of the coupons, which was measured in parallel by an established method. A new image analysis method was developed that utilizes multilevel thresholding and a ramp filter to measure biofilm growth intensity (BGI). Overall, this digital analysis assay was able to discern subtle differences in surface fouling that accumulated over time. The entirety of a surface of interest can be assessed with just one image, and this method is sensitive at the early stages of biofilm development.

Staining dramatically improved the contrast of subtle differences in biofilm accumulation and enabled a more detailed method of image analysis. Stains were specifically selected to highlight proteins and nucleic acids that make up the majority of fouling biomass. Broad application of this technique could take advantage of other stains to highlight different chemical and biochemical components. The combination of staining and advanced image analysis resulted in an assay that accurately quantifies early stages of biofilm formation. BGI data matched the independently measured cell density data with statistical correlation better than 0.9 (with 1 being a perfect match). All of the color channels analyzed with the BGI algorithm, including grayscale, were correlated with independent measurements of cell density, with the green channel and grayscale image resulting in the best fit. It is believed this is because the coupons used were green-tinted fiberglass, which changed in color with biofilm accumulation. The grayscale is an overall average of the individual color channels, and it is dominated by the contribution of the green channel in this case. Further study of subtle differences between the color channels may provide greater insight into the composition of surface growth. The resulting precision of this BGI quantification assay is similar to that of the more laborious means of measuring the optical density of suspended biofilm cultures.

This method could be used in field testing or other non-laboratory settings. The only special equipment needed is a digital camera and the stain. Analysis for this work was done with Matlab software on a personal computer, but it could also be implemented as a stand-alone program or even as a mobile application. Another advantage is that image analysis quantifies fouling over a relatively large surface area quickly and easily so an entire sample can be evaluated at once. In future work, the image analysis could be tailored to differentiate and compare regions in a single image. One potential challenge for field testing is uniform lighting, which is necessary for consistent comparisons between samples. Flat, smooth coupons were used in this study. Future experiments using textured coupons will determine if texture and shading impact the image analysis, particularly in the grayscale.

Another challenge is application of the stain: applications by pipette and dip rinsing are suitable for sample coupons, such as those used in several biofouling standard methods, but may prove challenging with larger structures. Other application techniques, such as spraying, could enable field testing of environmental biofilms on man-made marine structures and vessels. Future efforts should also include analysis of more complex fouling communities, such as those found in marine and dental biofilms. The BGI algorithm will allow cross sample comparisons, even with coatings of different colors, textures, and compositions.

## Electronic supplementary material

ESM 1 (PDF 903 kb)
