# Supplementary material for: A method for rapid quantitative assessment of biofilms with biomolecular staining and image analysis
Source: Anal Bioanal Chem. 2015 Dec 7;408:999–1008. doi: 10.1007/s00216-015-9195-z (PMC4709385; doi:10.1007/s00216-015-9195-z)
Supplement: Supplementary file 1 — (PDF 903 kb) [file 216_2015_9195_MOESM1_ESM.pdf]

Analytical and Bioanalytical Chemistry

Electronic Supplementary Material

A method for rapid quantitative assessment of biofilms with biomolecular staining and image analysis

Curtis Larimer, Eric Winder, Robert Jeters, Matthew Prowant, Ian Nettleship,
Raymond Shane Addleman, George T. Bonheyo

Table S1 Resolution and field of view of common biofilm imaging techniques

Method	Lateral resolution	Field of View	Limitations
**Digital Photography***	**5-10 μm limit**	**10 – 1000 mm**	**Requires even lighting for large samples**
Optical microscopy	<3.7 µm (330 nm limit)	<450 µm	Low resolution, small depth of field
Confocal microscopy	~250 nm limit	<450 µm<br>Slow, inaccurate raster for large areas	Large field of view requires slow inaccurate scanning
CLSM	~150 nm limit	<450 µm<br>Slow, inaccurate raster for large areas	Thin sample required, small field of view
Fluorescence microscopy	~250 nm limit	<450 µm	Sample must be fluorescent
Fluorescence super-resolution microscopy	~30 nm	1-2 µm	Sample must be fluorescent
Photoacoustic spectroscopy	100-200 µm	150 µm	Sample exposed to air
Ultrasonic imaging	~50 µm	> 1 mm (scanning)	Can't resolve low acoustic impedance differences
AFM (liquid)	<10 nm	~10 µm	Requires contact, weakly bound samples lower resolution
SEM	1-20 nm	~12 µm for high resolution	Destructive sample preparation
*Depends on camera sensor and lens configuration			

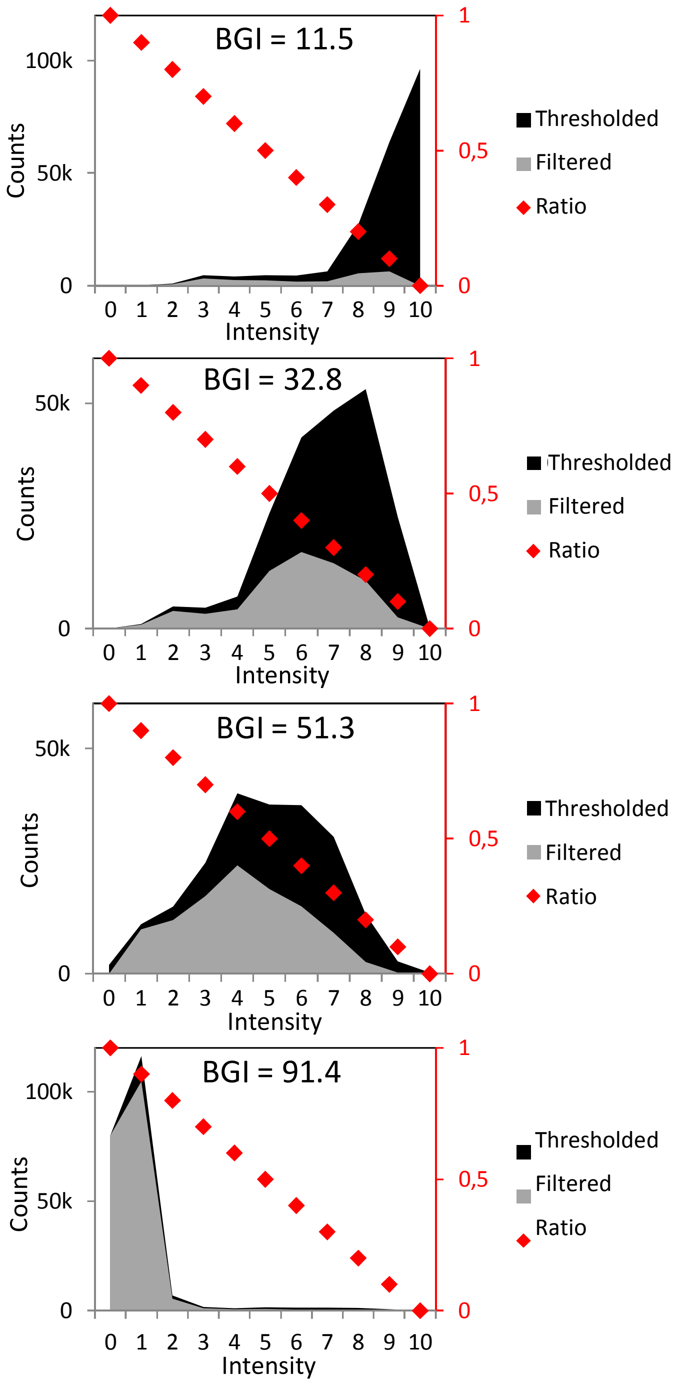

Figure S1 Examples of BGI calculation from histograms for a range of values. The histograms of multilevel thresholded images are shown in black. The gray histograms show the effect of a linear ramp filter (which is shown in red as the ratio of the two histograms). Images with many low intensity (darker) pixels result in higher BGI values

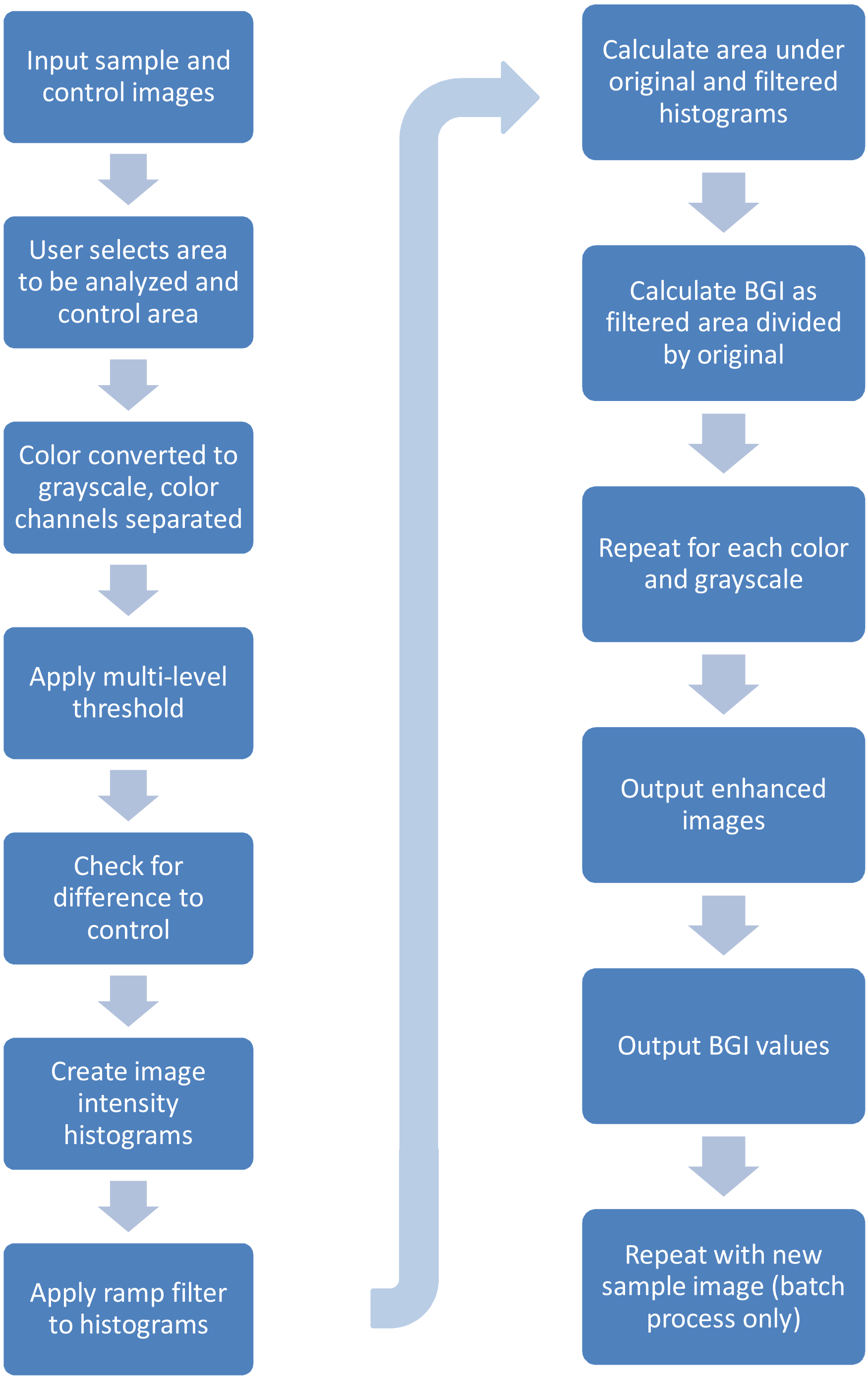

Figure S2 Flow diagram for BGI image analysis program

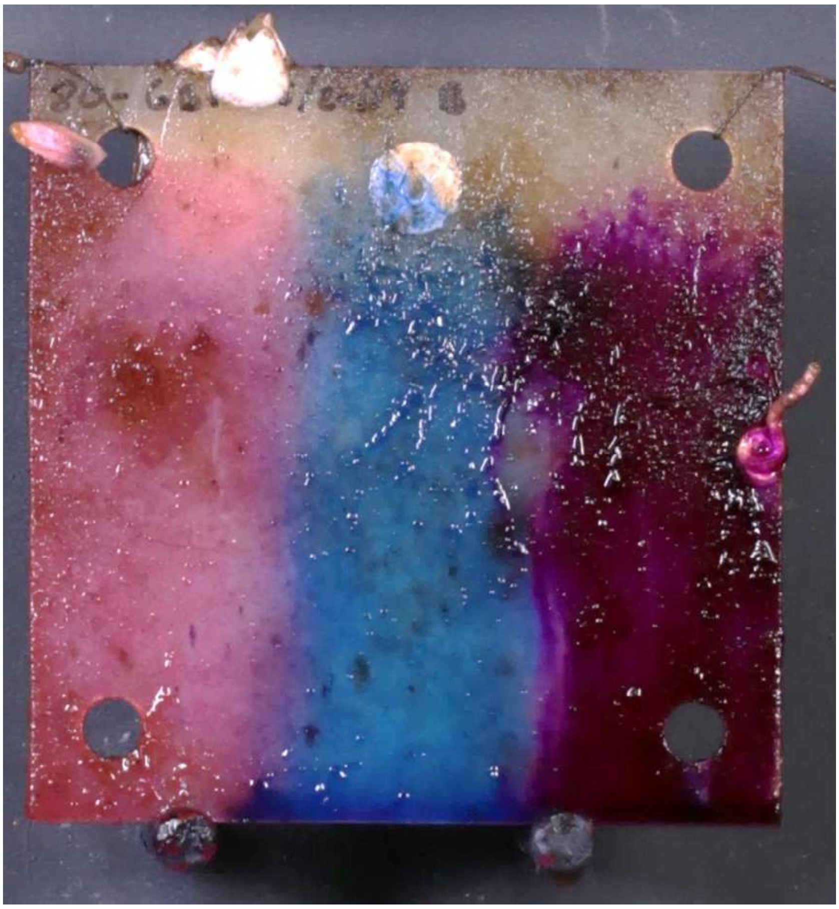

Figure S3 A coupon with three biomolecular stains applied. Erythrosine B (left), Coomassie Brilliant Blue (middle), and Rhodamine (right). When mixed and applied to a fouled coupon, this combination of stains attaches broadly to major components of biofouling

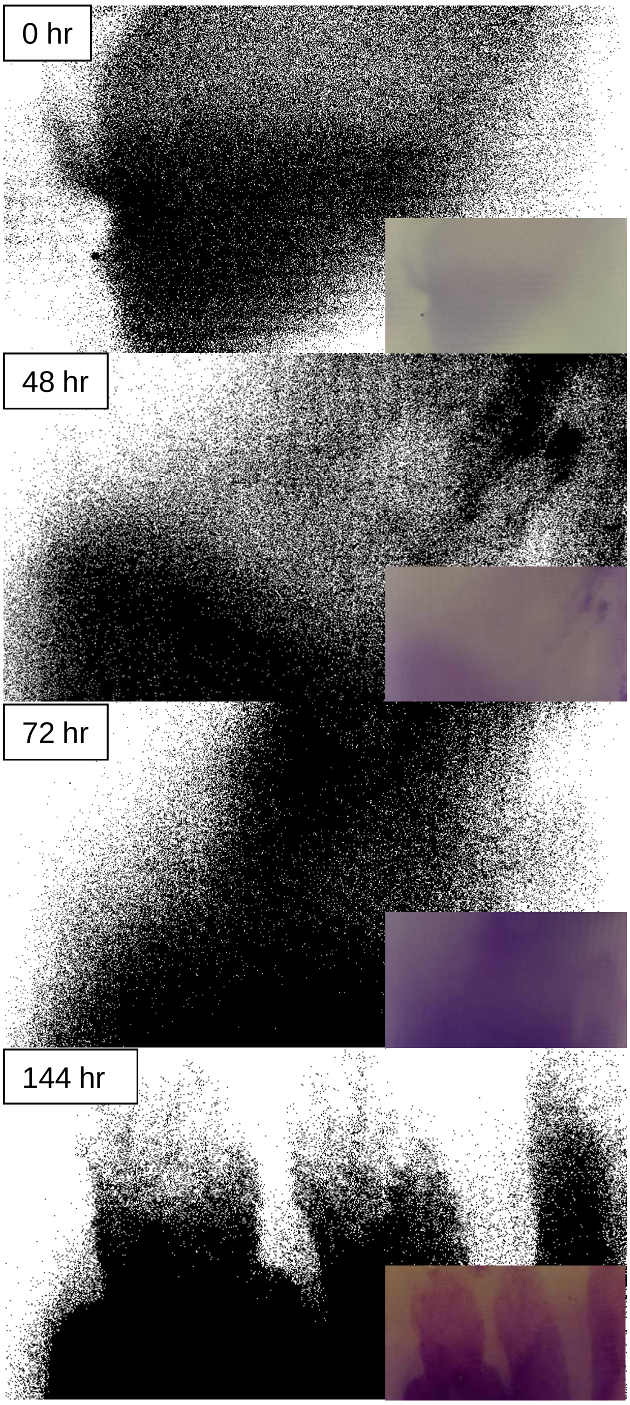

Figure S4 When images are analyzed with a bimodal thresholding technique like Otsu's method the distinction between time points is lost. In particular, the area covered by fouling (black) is overestimated at the earliest time point. For comparison the original color images are inset. The BGI method replaces this method with a more nuanced multilevel threshold in order to better quantify surface attached bacteria

Table S2 Statistical analysis of the correlation of various methods of image analysis to cell density. Best fit when correlation coefficient and slope are both nearest to 1. Color mapping of each cell shows relative fitness on the scale shown below. BGI data from stained images are highlighted with bold lettering. BGI data correlates strongly with cell density

	Unstained		Stained	
Method of analysis	Correlation coeff. (r)	Slope	Correlation coeff. (r)	Slope
Otsu gray	0.86	0.69	0.80	0.69
Otsu red	0.74	0.45	0.91	0.84
Otsu green	0.66	0.41	0.76	0.62
Otsu blue	0.81	0.68	0.96	0.85
Select gray	0.77	0.45	0.66	0.30
Select red	0.65	0.29	0.83	0.61
Select green	0.72	0.39	0.39	0.05
Select blue	0.27	0.00	0.77	0.46
BGI gray	0.46	0.32	**0.99**	**0.93**
BGI red	0.41	0.26	**0.97**	**0.87**
BGI green	0.69	0.59	**0.99**	**0.95**
BGI blue	0.00	0.00	**0.92**	**0.77**
Bad fit				Good fit

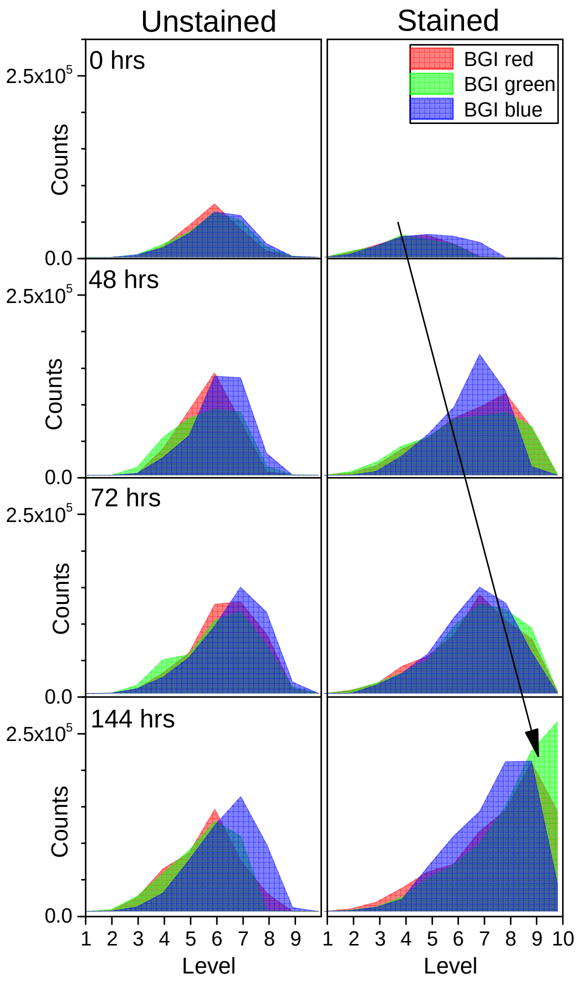

Figure S5 The BGI algorithm analyzes histograms from each color channel of digital photos. Here, BGI modified histograms for unstained and stained coupons are shown for each time point. Without the staining procedure there is not an obvious trend in the histograms over time. Data from stained coupons show a clear trend towards greater intensity levels with time. The shift is seen in each of the color channels (red, green, and blue)
